# Supplementary material for: Fecal Microbiota Transplantation in Decompensated Cirrhosis: A Systematic Review on Safety and Efficacy
Source: Antibiotics (Basel). 2022 Jun 23;11(7):838. doi: 10.3390/antibiotics11070838 (PMC9311594; doi:10.3390/antibiotics11070838)
Supplement: Supplementary file 1 [file antibiotics-11-00838-s001.zip › antibiotics-1772693-supplementary.pdf]

Supplemental Table S1: Search terms used to identify and filter studies from literature

Key words	Fecal microbiota transplant, cirrhosis
Inclusion	Any types of study with patient data and outcomes, any age, any sex
Exclusion	Animal studies , Single case reports
PubMed Search	("fecal microbiota transplantation" OR "faecal microbiota transplantation" OR "fecal microbiota transplant" OR "faecal microbiota transplant" OR "fecal microbiota transfer" OR "faecal microbiota transfer" OR "fecal transplant" OR "faecal transplant" OR "fecal transfer" OR "faecal transfer" OR "donor feces" OR "donor faeces" OR "donor stool" OR "bacteriotherapy" OR "FMT" OR "Fecal Microbiota Transplantation"[mh]) AND ("cirrhosis" OR "liver disease" OR "liver cirrhosis"[mh]) NOT animal
Filter	All studies
Filter	English

Supplemental Table S2: List of studies that were generated on initial literature search prior to screening

Author/Year	Title	Include/ Exclude
Yan 2021	Gut dysbiosis correction contributes to the hepatoprotective effects of Thymus quinquecostatus Celak extract against alcohol through the gut-liver axis	Exclude
Sun 2021	Myricetin supplementation decreases hepatic lipid synthesis and inflammation by modulating gut microbiota	Exclude
Matsui 2021	Ileal Bile Acid Transporter Inhibitor Improves Hepatic Steatosis	Exclude

Burz 2021	Fecal Microbiota Transplant from Human to Mice Gives Insights into the Role of the Gut Microbiota in Non-Alcoholic Fatty Liver Disease (NAFLD)	Exclude
Schneider 2020	Intestinal Dysbiosis Amplifies Acetaminophen-Induced Acute Liver Injury	Exclude
Wu 2020	Liver Injury Impaired 25-Hydroxylation of Vitamin D Suppresses Intestinal Paneth Cell defensins, leading to Gut Dysbiosis and Liver Fibrogenesis	Exclude
Jain 2021	Gut Microbiome: A Potential Modifiable Risk Factor in Biliary Atresia	Exclude
Saha 2020	Durability Of Response To Fecal Microbiota Transplantation After Exposure to Risk Factors for Recurrence In Patients With Clostridioides difficile Infection	Exclude
Xie 2019	Hair regrowth following fecal microbiota transplantation in an elderly patient with alopecia areata: A case report and review of the literature	Exclude
Sun 2019	The effect of fecal microbiota transplantation on Hepatic myelopathy: A case report	Exclude
Mullish 2018	Antibiotic-Associated Disruption of Microbiota Composition and Function in Cirrhosis Is Restored by Fecal Transplant	Exclude
Bajaj 2021	Distinct gut microbial compositional and functional changes associated with impaired inhibitory control in patients with cirrhosis	Exclude
Tarrago 2020	Combination of genetic polymorphisms in TLR influence cytokine profile in HCV patients treated with DAAs in the State of Amazonas	Exclude
Couto 2017	Prevalence and predictors for compensated Advanced Chronic Liver Disease (c-ACLD) in patients with chronic Hepatitis Delta Virus (HDV) infection	Exclude
Yang 2020	Potential role of intestinal microflora in disease progression among patients with different stages of Hepatitis B	Exclude
Gibiino 2021	The Other Side of Malnutrition in Inflammatory Bowel Disease (IBD): Non-Alcoholic Fatty Liver Disease	Exclude
Seong 2020	Fecal Microbiota Transplantation for multidrug-resistant organism: Efficacy and Response prediction	Exclude
Chen 2021	A botanical dietary supplement from white peony and licorice	Exclude
Arias 2019	Differences between diabetic and non-diabetic patients with community-acquired pneumonia in primary care in Spain	Exclude
Souza-Cruz 2016	Liver and blood cytokine microenvironment in HCV patients is associated to liver fibrosis score: a proinflammatory cytokine ensemble orchestrated by TNF and tuned by IL-10	Exclude

Davidovics 2019	Fecal Microbiota Transplantation for Recurrent Clostridium difficile Infection and Other Conditions in Children: A Joint Position Paper From the North American Society for Pediatric Gastroenterology, Hepatology, and Nutrition and the European Society for Pediatric Gastroenterology, Hepatology, and Nutrition	Exclude
Bubnov 2015	Probiotics and immunity: provisional role for personalized diets and disease prevention	Exclude
Patel 2015	New approaches for bacteriotherapy: prebiotics, new-generation probiotics, and synbiotics	Exclude
Colle 1989	Oral bacteriotherapy with Bifidobacterium bifidum and Lactobacillus acidophilus in cirrhotic patients	Exclude
Woodhouse 2019	PROFIT, a PROspective, randomised placebo controlled feasibility trial of Faecal mIcrobiota Transplantation in cirrhosis: study protocol for a single-blinded trial	Exclude
Allegretti 2019	Fecal Microbiota Transplantation in Patients With Primary Sclerosing Cholangitis: A Pilot Clinical Trial	Exclude
Bajaj 2019	Reply	Exclude
Philips 2019	Only in the darkness can you see the stars: Severe alcoholic hepatitis and higher grades of acute-on-chronic liver failure	Exclude
Bajaj 2018	Reply	Exclude
Craven 2021	Response to Ianiro et al	Exclude
Czarnecka 2021	Multidirectional facets of obesity management in the metabolic syndrome population after liver transplantation	Exclude
Philips 2019	Severe alcoholic hepatitis: current perspectives	Exclude
Lo 2019	The Transplantation of Fecal Microbiota for Cirrhotic Patients	Exclude
Campion 2019	Dietary approach and gut microbiota modulation for chronic hepatic encephalopathy in cirrhosis	Exclude
Alsahhar 2019	Updates on the pathophysiology and therapeutic targets for hepatic encephalopathy	Exclude
Alvarez-Mercado 2019	Microbial Population Changes and Their Relationship with Human Health and Disease	Exclude
Bajaj 2019	Alcohol, liver disease and the gut microbiota	Exclude
Nobili 2019	Fighting Fatty Liver Diseases with Nutritional Interventions, Probiotics, Symbiotics, and Fecal Microbiota Transplantation (FMT)	Exclude
Schwenger 2019	Non-alcoholic fatty liver disease and obesity: the role of the gut bacteria	Exclude
Gustot 2018	Acute-on-chronic liver failure vs. traditional acute decompensation of cirrhosis	Exclude

Delaune 2018	Fecal microbiota transplantation: a promising strategy in preventing the progression of non-alcoholic steatohepatitis and improving the anti-cancer immune response	Exclude
Vaughn 2019	Fecal Microbiota Transplantation: Current Status in Treatment of GI and Liver Disease	Exclude
Nardelli 2018	Management of Hepatic Encephalopathy Not Responsive to First-Line Treatments	Exclude
Zhang 2018	Microbiota transplantation: concept, methodology and strategy for its modernization	Exclude
Heath 2018	Fecal microbiota transplantation and its potential therapeutic uses in gastrointestinal disorders	Exclude
Aitbeav 2017	Liver diseases: The pathogenetic role of the gut microbiome and the potential of treatment for its modulation	Exclude
Bakker 2017	Fecal Microbiota Transplantation: Therapeutic Potential for a Multitude of Diseases beyond Clostridium difficile	Exclude
Tandon 2017	Fecal microbiota transplantation for hepatic encephalopathy: Ready for prime time?	Exclude
Arab 2018	Gut-liver axis, cirrhosis and portal hypertension: the chicken and the egg	Exclude
Kang 2017	Gut microbiota and hepatitis-B-virus-induced chronic liver disease: implications for faecal microbiota transplantation therapy	Exclude
Weiest 2017	Targeting the gut-liver axis in liver disease	Exclude
Sidhu 2017	The gut microbiome.	Exclude
Malikowski 2017	Fecal microbiota transplantation for gastrointestinal disorders	Exclude
Monsour 2016	The Microbiome: What Will the Future Hold?	Exclude
Heath 2016	Microbiome alterations observed in liver diseases present opportunities for potential fecal transplantation	Exclude
Haque 2016	Intestinal microbiota in liver disease	Exclude
Smits 2013	Therapeutic potential of fecal microbiota transplantation	Exclude
Weng 2019	Microbiota and gastrointestinal cancer	Exclude
Gustot 2019	Acute-on-chronic liver failure in patients with alcohol-related liver disease	Exclude
Bloom 2021	Microbiome Therapeutics for Hepatic Encephalopathy	Exclude
Nishikawa 2021	Dysbiosis and liver diseases (Review)	Exclude
Gu 2021	Clinical Application and Progress of Fecal Microbiota Transplantation in Liver Diseases: A Review	Exclude

Gupta 2021	Mechanistic and physiological approaches of fecal microbiota transplantation in the management of NAFLD	Exclude
Paratore 2021	Fecal Microbiota Transplantation in Patients with HBV Infection or Other Chronic Liver Diseases: Update on Current Knowledge and Future Perspectives	Exclude
Fianchi 2021	Nonalcoholic Fatty Liver Disease (NAFLD) as Model of Gut-Liver Axis Interaction: From Pathophysiology to Potential Target of Treatment for Personalized Therapy	Exclude
Bajaj 2021	The Evolving Challenge of Infections in Cirrhosis	Exclude
Ianiro 2021	Quantity of Donor Stool for Fecal Microbiota Transplantation: The More, the Better?	Exclude
Moreno-Gonzalez 2021	The Role of the Microbiome in Liver Cancer	Exclude
Hartmann 2021	New Developments in Microbiome in Alcohol-associated and Non-alcoholic Fatty Liver Disease	Exclude
Hrncir 2021	Gut Microbiota and NAFLD: Pathogenetic Mechanisms, Microbiota Signatures, and Therapeutic Interventions	Exclude
Moon 2021	Fecal Microbiota Transplantation beyond Clostridioides Difficile Infection	Exclude
Coskun 2021	Therapeutic modulation methods of gut microbiota and gut-liver axis	Exclude
Bovi 2021	Oxidative Stress in Non-alcoholic Fatty Liver Disease. An Updated Mini Review	Exclude
Afecto 2021	Fecal microbiota transplantation in hepatic encephalopathy : a review of the current evidence and future perspectives.	Exclude
Goldenberg 2021	The role of faecal microbiota transplantation: looking beyond Clostridioides difficile infection	Exclude
Mullish 2021	The gut microbiome: what every gastroenterologist needs to know	Exclude
Gawlik-Kotelnicka 2021	Adiposity in Depression or Depression in Adiposity? The Role of Immune-Inflammatory-Microbial Overlap	Exclude
Plaza-Diaz 2021	Insights into the Impact of Microbiota in the Treatment of NAFLD/NASH and Its Potential as a Biomarker for Prognosis and Diagnosis	Exclude
Hassouneh 2021	Gut Microbiota Modulation and Fecal Transplantation: An Overview on Innovative Strategies for Hepatic Encephalopathy Treatment	Exclude
Dai 2020	Microbial Metabolites: Critical Regulators in NAFLD	Exclude
Sehgal 2020	Role of Microbiota in Pathogenesis and Management of Viral Hepatitis	Exclude

Song 2020	Medical Treatment of Alcoholic Liver Disease	Exclude
Chen 2020	Gut Microbiota Metabolites in NAFLD Pathogenesis and Therapeutic Implications	Exclude
Reuter 2020	Microbiome: Emerging Concepts in Patients with Chronic Liver Disease	Exclude
Shasthry 2020	Fecal microbiota transplantation in alcohol related liver diseases	Exclude
Iruzubieta 2020	A Role for Gut Microbiome Fermentative Pathways in Fatty Liver Disease Progression	Exclude
Alimirah 2020	Novel Therapies in Hepatic Encephalopathy	Exclude
Khoruts 2020	Reply to: " 'You know my name, but not my story' - Deciding on an accurate nomenclature for faecal microbiota transplantation": Intestinal microbiota transplantation: Naming a new paradigm	Exclude
Philips 2020	You know my name, but not my story' - Deciding on an accurate nomenclature for faecal microbiota transplantation	Exclude
Veryan 2019	Recent advances in alcoholic hepatitis	Exclude
Lechner 2020	Fecal Microbiota Transplantation for Chronic Liver Diseases: Current Understanding and Future Direction	Exclude
Bajaj 2020	Microbiota changes and intestinal microbiota transplantation in liver diseases and cirrhosis	Exclude
Gerussi 2020	Multiple therapeutic targets in rare cholestatic liver diseases: Time to redefine treatment strategies	Exclude
Li 2019	Microbiome dysbiosis and alcoholic liver disease	Exclude
Shah 2020	Targeting the Gut Microbiome as a Treatment for Primary Sclerosing Cholangitis: A Conceptional Framework	Exclude
Wang 2019	Advances in Gut Microbiota of Viral Hepatitis Cirrhosis	Exclude
Cold 2021	Systematic review with meta-analysis: encapsulated faecal microbiota transplantation - evidence for clinical efficacy	Exclude
Chen 2021	The role of gut microbiota in hepatitis B disease progression and treatment	Exclude
Bayoumy 2021	Gut fermentation syndrome: A systematic review of case reports	Exclude
Madsen 2021	Fecal microbiota transplantation in hepatic encephalopathy: a systematic review	Exclude
Sharpton 2019	Gut microbiome-targeted therapies in nonalcoholic fatty liver disease: a systematic review, meta-analysis, and meta-regression	Exclude
Rodriguez 2017	Emerging treatments for primary sclerosing cholangitis	Exclude

Meighani 2020	Fecal Microbiota Transplantation for Clostridioides Difficile Infection in Patients with Chronic Liver Disease	Fully reviewed and excluded
Bajaj 2019	Microbial functional change is linked with clinical outcomes after capsular fecal transplant in cirrhosis	Fully reviewed and excluded
Craven 2020	Allogenic Fecal Microbiota Transplantation in Patients With Nonalcoholic Fatty Liver Disease Improves Abnormal Small Intestinal Permeability: A Randomized Control Trial	Fully reviewed and excluded
Bajaj 2020	Fecal Microbiota Transplant in Cirrhosis Reduces Gut Microbial Antibiotic Resistance Genes: Analysis of Two Trials	Fully reviewed and excluded
Bajaj 2021	A Randomized Clinical Trial of Fecal Microbiota Transplant for Alcohol Use Disorder	Fully reviewed and excluded
Bajaj 2018	Antibiotic-Associated Disruption of Microbiota Composition and Function in Cirrhosis Is Restored by Fecal Transplant	Fully reviewed and excluded
Philips 2018	Corticosteroids, nutrition, pentoxifylline, or fecal microbiota transplantation for severe alcoholic hepatitis	Fully reviewed and excluded
Pringle 2019	Patients With Cirrhosis Require More Fecal Microbiota Capsules to Cure Refractory and Recurrent Clostridium difficile Infections	Fully reviewed and excluded
Bajaj 2019	Long-term Outcomes of Fecal Microbiota Transplantation in Patients With Cirrhosis	Fully reviewed and excluded
Bajaj 2019	Fecal Microbial Transplant Capsules Are Safe in Hepatic Encephalopathy: A Phase 1, Randomized, Placebo-Controlled Trial	Include
Cheng 2020	Fecal Microbiota Transplantation Is Safe and Effective in Patients With Clostridioides difficile Infection and Cirrhosis	Include
Bajaj 2017	Fecal microbiota transplant from a rational stool donor improves hepatic encephalopathy: A randomized clinical trial	Include
Mehta 2018	Preliminary experience with single fecal microbiota transplant for treatment of recurrent overt hepatic encephalopathy-A case series	Include
Olmedo 2019	Is it reasonable to perform Fecal Microbiota Transplantation for recurrent Clostridium difficile Infection in patients with liver cirrhosis?	Include

Supplemental Table S3: Risk of bias assessment for Bajaj 2017 [8]

Responses underlined in green are potential markers for low risk of bias, and responses in red are potential markers for a risk of bias. Where questions relate only to sign posts to other questions, no formatting is used.

Domain 1: Risk of bias arising from the randomization process

Signalling questions	Response options
**1.1 Was the allocation sequence random?**	Y / PY / PN / N / NI
**1.2 Was the allocation sequence concealed until participants were enrolled and assigned to interventions?**	Y / PY / PN / N / NI
**1.3 Did baseline differences between intervention groups suggest a problem with the randomization process?**	Y / PY / PN / N / NI
**Risk-of-bias judgement**	Low / High / Some concerns

Domain 2: Risk of bias due to deviations from the intended interventions (*effect of assignment to intervention*)

Signalling questions	Response options
**2.1. Were participants aware of their assigned intervention during the trial?**	Y / PY / PN / N / NI
**2.2. Were carers and people delivering the interventions aware of participants' assigned intervention during the trial?**	Y / PY / PN / N / NI
**2.3. If Y/PY/NI to 2.1 or 2.2: Were there deviations from the intended intervention that arose because of the trial context?**	NA / Y / PY / PN / N / NI
**2.4 If Y/PY to 2.3: Were these deviations likely to have affected the outcome?**	NA / Y / PY / PN / N / NI
**2.5. If Y/PY/NI to 2.4: Were these deviations from intended intervention balanced between groups?**	NA / Y / PY / PN / N / NI
**2.6 Was an appropriate analysis used to estimate the effect of assignment to intervention?**	Y / PY / PN / N / NI

**2.7 If N/PN/NI to 2.6: Was there potential for a substantial impact (on the result) of the failure to analyse participants in the group to which they were randomized?**	NA / Y / PY / PN / N / NI
**Risk-of-bias judgement**	Low / High / Some concerns

Domain 2: Risk of bias due to deviations from the intended interventions (*effect of adhering to intervention*)

Signalling questions	Response options
**2.1. Were participants aware of their assigned intervention during the trial?**	Y / PY / PN / N / NI
**2.2. Were carers and people delivering the interventions aware of participants' assigned intervention during the trial?**	Y / PY / PN / N / NI
**2.3. [If applicable:] If Y/PY/NI to 2.1 or 2.2: Were important non-protocol interventions balanced across intervention groups?**	NA / Y / PY / PN / N / NI
**2.4. [If applicable:] Were there failures in implementing the intervention that could have affected the outcome?**	NA / Y / PY / PN / N / NI
**2.5. [If applicable:] Was there non-adherence to the assigned intervention regimen that could have affected participants' outcomes?**	NA / Y / PY / PN / N / NI
**2.6. If N/PN/NI to 2.3, or Y/PY/NI to 2.4 or 2.5: Was an appropriate analysis used to estimate the effect of adhering to the intervention?**	NA / Y / PY / PN / N / NI
**Risk-of-bias judgement**	Low / High / Some concerns
Optional: What is the predicted direction of bias due to deviations from intended interventions?	NA / Favours experimental / Favours comparator / Towards null /Away from null / Unpredictable

Domain 3: Missing outcome data

Signalling questions	Response options
**3.1 Were data for this outcome available for all, or nearly all, participants randomized?**	Y / PY / PN / N / NI

**3.2 If N/PN/NI to 3.1: Is there evidence that the result was not biased by missing outcome data?**	NA / Y / PY / PN / N
**3.3 If N/PN to 3.2: Could missingness in the outcome depend on its true value?**	NA / Y / PY / PN / N / NI
**3.4 If Y/PY/NI to 3.3: Is it likely that missingness in the outcome depended on its true value?**	NA / Y / PY / PN / N / NI
**Risk-of-bias judgement**	Low / High / Some concerns

Domain 4: Risk of bias in measurement of the outcome

**Signalling questions**	**Response options**
**4.1 Was the method of measuring the outcome inappropriate?**	Y / PY / PN / N / NI
**4.2 Could measurement or ascertainment of the outcome have differed between intervention groups?**	Y / PY / PN / N / NI
**4.3 If N/PN/NI to 4.1 and 4.2: Were outcome assessors aware of the intervention received by study participants?**	NA / Y / PY / PN / N / NI
**4.4 If Y/PY/NI to 4.3: Could assessment of the outcome have been influenced by knowledge of intervention received?**	NA / Y / PY / PN / N / NI
**4.5 If Y/PY/NI to 4.4: Is it likely that assessment of the outcome was influenced by knowledge of intervention received?**	NA / Y / PY / PN / N / NI
**Risk-of-bias judgement**	Low / High / Some concerns

Domain 5: Risk of bias in selection of the reported result

**Signalling questions**	**Response options**
**5.1 Were the data that produced this result analysed in accordance with a pre-specified analysis plan that was finalized before unblinded outcome data were available for analysis?**	Y / PY / PN / N / NI
**Is the numerical result being assessed likely to have been selected, on the basis of the results, from...**	

**5.2. ... multiple eligible outcome measurements (e.g. scales, definitions, time points) within the outcome domain?**	Y / PY / PN / N / NI
**5.3 ... multiple eligible analyses of the data?**	Y / PY / PN / N / NI
**Risk-of-bias judgement**	Low / High / Some concerns

Overall risk of bias

**Risk-of-bias judgement**	Low / High / Some concerns

Supplemental Table S4: Risk of bias assessment for Bajaj 2019 [9]

Responses underlined in green are potential markers for low risk of bias, and responses in red are potential markers for a risk of bias. Where questions relate only to sign posts to other questions, no formatting is used.

Domain 1: Risk of bias arising from the randomization process

Signalling questions	Response options
**1.1 Was the allocation sequence random?**	Y / PY / PN / N / NI
**1.2 Was the allocation sequence concealed until participants were enrolled and assigned to interventions?**	Y / PY / PN / N / NI
**1.3 Did baseline differences between intervention groups suggest a problem with the randomization process?**	Y / PY / PN / N / NI

**Risk-of-bias judgement**	Low / High / Some concerns

Domain 2: Risk of bias due to deviations from the intended interventions (*effect of assignment to intervention*)

**Signalling questions**	**Response options**
**2.1. Were participants aware of their assigned intervention during the trial?**	Y / PY / PN / N / NI
**2.2. Were carers and people delivering the interventions aware of participants' assigned intervention during the trial?**	Y / PY / PN / N / NI
**2.3. If Y/PY/NI to 2.1 or 2.2: Were there deviations from the intended intervention that arose because of the trial context?**	NA / Y / PY / PN / N / NI
**2.4 If Y/PY to 2.3: Were these deviations likely to have affected the outcome?**	NA / Y / PY / PN / N / NI
**2.5. If Y/PY/NI to 2.4: Were these deviations from intended intervention balanced between groups?**	NA / Y / PY / PN / N / NI
**2.6 Was an appropriate analysis used to estimate the effect of assignment to intervention?**	Y / PY / PN / N / NI
**2.7 If N/PN/NI to 2.6: Was there potential for a substantial impact (on the result) of the failure to analyse participants in the group to which they were randomized?**	NA / Y / PY / PN / N / NI
**Risk-of-bias judgement**	Low / High / Some concerns

Domain 2: Risk of bias due to deviations from the intended interventions (*effect of adhering to intervention*)

**Signalling questions**	**Response options**
**2.1. Were participants aware of their assigned intervention during the trial?**	Y / PY / PN / N / NI
**2.2. Were carers and people delivering the interventions aware of participants' assigned intervention during the trial?**	Y / PY / PN / N / NI
**2.3. [If applicable:] If Y/PY/NI to 2.1 or 2.2: Were important non-protocol interventions balanced across intervention groups?**	NA / Y / PY / PN / N / NI
**2.4. [If applicable:] Were there failures in implementing the intervention that could have affected the outcome?**	NA / Y / PY / PN / N / NI

**2.5. [If applicable:] Was there non-adherence to the assigned intervention regimen that could have affected participants' outcomes?**	NA / Y / PY / PN / N / NI
**2.6. If N/PN/NI to 2.3, or Y/PY/NI to 2.4 or 2.5: Was an appropriate analysis used to estimate the effect of adhering to the intervention?**	NA / Y / PY / PN / N / NI
**Risk-of-bias judgement**	Low / High / Some concerns
Optional: What is the predicted direction of bias due to deviations from intended interventions?	NA / Favours experimental / Favours comparator / Towards null /Away from null / Unpredictable

Domain 3: Missing outcome data

Signalling questions	Response options
**3.1 Were data for this outcome available for all, or nearly all, participants randomized?**	Y / PY / PN / N / NI
**3.2 If N/PN/NI to 3.1: Is there evidence that the result was not biased by missing outcome data?**	NA / Y / PY / PN / N
**3.3 If N/PN to 3.2: Could missingness in the outcome depend on its true value?**	NA / Y / PY / PN / N / NI
**3.4 If Y/PY/NI to 3.3: Is it likely that missingness in the outcome depended on its true value?**	NA / Y / PY / PN / N / NI
**Risk-of-bias judgement**	Low / High / Some concerns

Domain 4: Risk of bias in measurement of the outcome

Signalling questions	Response options
**4.1 Was the method of measuring the outcome inappropriate?**	Y / PY / PN / N / NI

**4.2 Could measurement or ascertainment of the outcome have differed between intervention groups?**	Y / PY / PN / N / NI
**4.3 If N/PN/NI to 4.1 and 4.2: Were outcome assessors aware of the intervention received by study participants?**	NA / Y / PY / PN / N / NI
**4.4 If Y/PY/NI to 4.3: Could assessment of the outcome have been influenced by knowledge of intervention received?**	NA / Y / PY / PN / N / NI
**4.5 If Y/PY/NI to 4.4: Is it likely that assessment of the outcome was influenced by knowledge of intervention received?**	NA / Y / PY / PN / N / NI
**Risk-of-bias judgement**	Low / High / Some concerns

Domain 5: Risk of bias in selection of the reported result

**Signalling questions**	**Response options**
**5.1 Were the data that produced this result analysed in accordance with a pre-specified analysis plan that was finalized before unblinded outcome data were available for analysis?**	Y / PY / PN / N / NI
**Is the numerical result being assessed likely to have been selected, on the basis of the results, from...**	
**5.2. ... multiple eligible outcome measurements (e.g. scales, definitions, time points) within the outcome domain?**	Y / PY / PN / N / NI
**5.3 ... multiple eligible analyses of the data?**	Y / PY / PN / N / NI
**Risk-of-bias judgement**	Low / High / Some concerns

Overall risk of bias

**Risk-of-bias judgement**	Low / High / Some concerns

Supplemental Table S5: Risk of bias assessment of case series

Study	Mehta et al., 2018	Olmedo et al., 2020
1. Was the study question or objective clearly stated?	Y	Y
2. Was the study population clearly and fully described, including a case definition?	Y	Y
3. Were the cases consecutive?	Y	Y
4. Were the subjects comparable?	N	N
5. Was the intervention clearly described?	Y	Y
6. Were the outcome measures clearly defined, valid, reliable, and implemented consistently across all study participants?	Y	Y
7. Was the length of follow-up adequate?	Y	Y
8. Were the statistical methods well-described?	N	Y
9. Were the results well-described?	Y	Y
Total Quality score (total number of "yes"	7	8

Quality (7-9 yes: Good; 4-6 yes: Fair; 1-3 yes: Poor)	Good	Good

-

Supplemental Table 6: Risk of bias assessment of observational cohort studies

Study	Cheng et al., 2021
1. Was the study question or objective clearly stated?	Y
2. Was the study population clearly and fully described?	Y
3. Was the participation rate of eligible persons at least 50%?	Not applicable
4. Were all the subjects selected or recruited from the same or similar populations (including the same time period)? Were inclusion and exclusion criteria for being in the study prespecified and applied uniformly to all participants?	Y
5. Was a sample size justification, power description, or variance and effect estimates provided?	N
6. For the analyses in this paper, were the exposure(s) of interest measured prior to the outcome(s) being measured?	Y
7. Was the timeframe sufficient so that one could reasonably expect to see an association between exposure and outcome if it existed?	Y

**8. For exposures that can vary in amount or level, did the study examine different levels of the exposure as related to the outcome (e.g., categories of exposure, or exposure measured as continuous variable)?**	**Y**
**9. Were the exposure measures (independent variables) clearly defined, valid, reliable, and implemented consistently across all study participants?**	**Y**
**10. Was the exposure(s) assessed more than once over time?**	**N**
**11. Were the outcome measures (dependent variables) clearly defined, valid, reliable, and implemented consistently across all study participants?**	**Y**
**12. Were the outcome assessors blinded to the exposure status of participants?**	**Not Applicable**
**13. Was loss to follow-up after baseline 20% or less?**	**Not Applicable**
**14. Were key potential confounding variables measured and adjusted statistically for their impact on the relationship between exposure(s) and outcome(s)?**	**Y**
**Total Quality score (total number of "yes"**	**9**
**Quality (7-9 yes: Good; 4-6 yes: Fair; 1-3 yes: Poor)**	**Good**
